# 造血干细胞移植后难治性/耐药性巨细胞病毒感染诊疗进展

**DOI:** 10.3760/cma.j.cn121090-20240615-00223

**Published:** 2024-11

**Authors:** 潇予 张, 尔烈 姜

**Affiliations:** 1 中国医学科学院血液病医院（中国医学科学院血液学研究所）北京协和医学院，血液与健康全国重点实验室，国家血液系统疾病临床医学研究中心，天津市血液病细胞治疗研究重点实验室，细胞生态海河实验室，天津 300020 State Key Laboratory of Experimental Hematology, National Clinical Research Center for Blood Diseases, Tianjin Key Laboratory of Cell Therapy for Blood Diseases, Haihe Laboratory of Cell Ecosystem, Institute of Hematology & Blood Diseases Hospital, Chinese Academy of Medical Sciences & Peking Union Medical College, Tianjin 300020, China; 2 天津医学健康研究院，天津 301600 Tianjin Institutes of Health Science, Tianjin 301600, China

## Abstract

巨细胞病毒（CMV）感染是造血干细胞移植（HSCT）后最常见的机会性感染之一。尽管预防和抢先治疗可以降低CMV感染和CMV病的发生率，但仍有部分患者发展为难治性/耐药性（R/R）CMV感染，严重影响预后。对抗CMV治疗药物不耐受和发生耐药是患者发展为难治的重要原因。在CMV DNA定量PCR的基础上联合使用CMV特异性免疫介导（CMI）监测可以优化R/R CMV感染的诊断并指导个性化治疗。近年来，新型抗病毒药物、免疫治疗包括免疫球蛋白和病毒特异性T细胞治疗的探索，有望为该类患者提供更多的治疗选择。

## Abstract (en)

Cytomegalovirus（CMV）infection is one of the most prevalent opportunistic infections after hematopoietic stem cell transplantation（HSCT）. Prophylaxis and preemptive therapy have demonstrated promise in reducing the incidence of CMV infection and CMV disease, but the management of refractory/resistant（R/R）CMV infections after HSCT remains a challenge that significantly affects the prognosis of patients undergoing HSCT. Intolerance and resistance to antivirals are the primary reasons for developing refractory CMV infections. CMV DNA quantification PCR combined with CMV-specific cell-mediated immunity monitoring may help to optimize diagnosis and enable personalized management of R/R CMV infection. Novel antiviral drugs and other immunotherapies, including intravenous immunoglobulin and adoptive CMV T cell therapy, constitute an appealing option.

造血干细胞移植（hematopoietic stem cell transplantation，HSCT）是恶性血液肿瘤、骨髓衰竭性疾病等多种血液系统疾病的重要治疗方案。然而，HSCT后各类感染的风险显著增加，其中巨细胞病毒（cytomegalovirus，CMV）感染发生率较高，是常见的感染之一[1]–[2]。文献报道我国人群中CMV血清阳性率约为90％[3]。鉴于供者及受者CMV阳性率均高，致使HSCT受者移植后CMV激活发生率达30％～70％[4]。而CMV终末器官疾病是造成移植后患者死亡的主要原因之一。普遍性预防以及抢先治疗在一定程度上已降低了CMV感染和CMV病的发生率，但HSCT后难治性/耐药性（refractory/resistant，R/R）CMV感染仍是治疗的难点[5]。近年来，诊断手段的进展及新型药物获批使得R/R CMV感染患者获益。

一、HSCT后CMV感染概况

CMV是一种属于疱疹病毒目乙型疱疹病毒亚科的双链DNA病毒，又称疱疹病毒5型，在普通人群中分布广泛，血清阳性率为24.6％～95.7％[3],[6]。供者及受者的高CMV阳性率使得HSCT后CMV激活发生率增加，既往临床数据显示HSCT受者移植后CMV激活发生率达30％～70％，是HSCT后最常见的感染之一[4]。CMV感染可导致CMV病、急慢性移植物抗宿主病（graft-versus-host disease, GVHD）以及骨髓抑制等，影响移植患者的预后[7]。

二、R/R CMV感染的定义

CMV感染对抗病毒药物治疗无反应的现象被统称为R/R CMV感染[8]。既往难治性CMV感染指接受合理的抗病毒治疗2周后CMV病毒载量维持（即≤log 10的CMV DNA水平升高或降低）或上升（即>log 10的CMV DNA水平升高），症状、体征无改善或持续进展；然而部分移植后CMV感染患者出现骨髓抑制或肾损伤，继而无法耐受抗CMV治疗，这部分患者在临床实践中也可纳为难治性CMV感染。

耐药性CMV感染指在符合难治性CMV诊断的基础上，有特定的基因突变导致CMV对一种或多种治疗药物的敏感性下降。

三、HSCT后R/R CMV感染的发生率及风险因素

虽然CMV预防治疗可以一定程度降低HSCT后R/R CMV感染发生风险[9]，但仍有一定比例HSCT受者会发生R/R CMV感染。全球范围内，据报道HSCT受者耐药性CMV感染的发生率为1.7％～14.5％，难治性CMV感染发生率为29％～39％[10]–[11]。中国HSCT后难治性CMV感染发生率略高于全球水平，约为47％[12]–[13]。

R/R CMV感染发生的风险因素和患者发展为难治的原因包括宿主相关因素、病毒相关因素以及抗病毒药物相关因素。宿主相关因素包括长期抗病毒治疗、既往抗CMV病毒药物暴露、复发性感染、药物吸收或转化不良、去T细胞移植、免疫重建延迟、单倍体或脐血HSCT、供者CMV血清学阴性/受者CMV血清学阳性（D-/R+）、活动状态GVHD及免疫抑制治疗[8],[14]。在中国HSCT后R/R CMV患者群体相关研究中，Bao等[15]单中心回顾性研究显示，单倍体HSCT、脐血HSCT、全身照射预处理以及大剂量糖皮质激素暴露（≥1 mg·kg^−1^·d^−1^）是难治性CMV感染的风险因素。2021年Liu等[16]在供者CMV血清学阴性状态对HSCT患者预后影响的研究中也证实，D-/R+患者群体难治性CMV感染发生率较高。2022年Duan等[17]研究显示血小板减少和抗胸腺细胞球蛋白（antithymocyte globulin, ATG）暴露都会增加难治性CMV感染发生率。

Nho等[18]开展的真实世界研究中纳入306例HSCT患者，其中有18例（15.9％）患者在HSCT后发展为R/R CMV感染，这18例患者均发生GVHD。中国单倍体HSCT的比例较高可能是难治性CMV感染发生率高于全球水平的潜在原因[19]。Shen等[12]建立了预测单倍体HSCT后R/R CMV感染的模型，有助于对患者进行早期的危险分层，同时还可以预测HSCT后的死亡率和存活率。

病毒相关因素包括高病毒载量、抗病毒治疗期间（>2周足量药物）病毒载量升高、间歇性低水平CMV血症、接受抗病毒治疗CMV血症首次缓解后病毒载量再升高、CMV感染复发、CMV血症水平波动[10],[14],[20]。Allice等[21]的研究纳入13例HSCT后CMV高病毒载量（>1×10^4^拷贝数/ml）并存在CMV感染复发的患者，结果显示这类患者R/R CMV感染发生风险高。Duan等[17]的研究结果中也证实较高的病毒载量（>1×10^4^拷贝数/ml）与难治性CMV感染发生风险相关。

抗病毒药物相关因素主要指患者对抗CMV治疗药物不耐受和发生耐药。一项系统综述分析了HSCT受者传统治疗相关的严重不良事件，使用更昔洛韦（GCV）的患者骨髓抑制发生率为10.0％，中性粒细胞减少发生率为30.0％～39.8％，此外约11％的患者发生肾毒性，高达13.6％的患者因此中断治疗[22]。GCV/缬更昔洛韦（VGCV）相关的骨髓抑制毒性可直接影响细胞毒性T淋巴细胞（cytotoxic T-lymphocyte，CTL）的产生、延缓CTL相关的免疫重建、增加晚期CMV感染的风险，在缺乏细胞免疫的情况下，GCV/VGCV抗CMV的疗效有限。同时，骨髓抑制引起的粒细胞减少可能增加其他机会性感染风险，血小板减少会增加患者出血风险、甚至有致命性出血发生的可能。膦甲酸钠（FOS）相关的肾毒性可能会引起急性肾损伤，严重时可能引起肾衰竭。因此，患者对药物不耐受而导致的用药剂量调整、停药均可能引起抗病毒治疗药物的耐药、CMV感染复发、病毒清除时间延长，增加CMV病发生风险[23]。

四、R/R CMV感染的诊断

难治性CMV感染诊断的基础仍是抗病毒治疗后CMV病毒载量，外周血样本中CMV DNA定量PCR（qPCR）是诊断金标准，但由于检测方法尚未统一，DNA的提取方法及样本类型均与检测结果差异性相关[24]–[25]，因此，指南建议使用同一样本类型及检测方法以保证结果的可靠性[26]–[27]，新方法的应用例如液滴数字PCR有望改善检测结果差异[28]。当CMV感染终末器官时，血样本CMV DNA检测敏感性下降[29]，因此在特定CMV病（例如CMV胃肠道炎、CMV肺炎、CMV脑炎）中，组织体液标本（例如胃肠道活检标本、粪便样本、支气管肺泡灌洗液、脑脊液）进行CMV检测均为目前可行的诊断方法[30]–[32]。由于qPCR检测较为敏感，仅基于病毒载量选择的抗病毒治疗方案可能导致长期和不必要的药物暴露，因此可以考虑合并使用CMV特异性免疫介导（CMV-specific cell mediated immunity, CMI）监测以优化诊断[33]。目前可用的CMI试剂盒包括QuantiFERON-CMV（QFN-CMV）、T-Track和T-Spot.CMV，其原理为检测CMV特异性抗原或肽刺激CD4^+^和（或）CD8^+^ T细胞释放的γ干扰素水平[34]–[35]。Thompson等[36]的研究中在HSCT后第100天和第150天应用QFN-CMV监测CMV再激活，QFN-CMV第100天的敏感性为62％，特异性为100％，阳性及阴性预测值分别为100％及39％。Wißkirchen等[37]在HSCT后使用T-Track监测CMV再激活，阴性预测值为92％。联合使用CMV qPCR与CMI试剂盒可以预测新发和复发CMV病的风险，监测移植患者的抗CMV免疫水平，指导治疗停药时机，个性化管理患者治疗方案[35]。

耐药性CMV感染的诊断依赖基因型检测，目前二代测序（NGS）技术可以对CMV进行快速、可靠、灵敏的基因型检测。上一代测序技术存在的局限性，例如对<10 000 U/ml的CMV拷贝数的灵敏度有限，可能存在假阳性，在没有表型证实的情况下，耐药性突变无法与多态性区分。NGS检测的引入有望克服这一限制[38]。Lodding等[39]的研究回顾性纳入20例UL97和UL54突变耐药性CMV感染患者，NGS能识别出传统测序识别出的所有耐药性基因突变位点，并在8/13（62％）的患者中识别出了新的UL97和UL54耐药性突变位点。

五、R/R CMV感染的治疗

（一）R/R CMV感染治疗现状

HSCT后R/R CMV感染治疗的药物选择有限，目前常用药物为GCV、VGCV、FOS及西多福韦（CDV）[10]。对于经典CMV一线治疗用药，既往文献报道FOS单药或联合用药［包括CMV免疫球蛋白（IVIG）、CDV、GCV］治疗R/R CMV感染，1年死亡率仍高达41％，CMV病的发生率约24％[10]。最近的一项真实世界研究中期分析结果显示[40]，使用以上药物治疗R/R CMV感染的患者中，分别有35.5％和25.8％产生骨髓抑制和肾毒性，可见目前R/R CMV治疗方案存在一定的局限性。针对R/R CMV感染，患者迫切需要病毒清除率高、清除速度快、安全性好的治疗方案。

（二）R/R CMV新型药物及在研化合物

1. 抗病毒治疗：

（1）马立巴韦（MBV）：MBV是一种新的苯并咪唑核糖苷类药物，与传统抗病毒药物进入感染细胞抑制病毒DNA合成不同，具有全新的抗CMV作用机制。MBV是一种CMV UL97蛋白激酶抑制剂，不仅能抑制病毒DNA合成，同时还可以抑制病毒衣壳化、抑制核衣壳出口，具有多模式抗CMV活性[41]。体外研究显示MBV对EB病毒也具有抗病毒活性[42]。MBV的半数效应浓度（EC_50_）为0.3 µmol/L，其对CMV的效力为GCV的10倍[43]。MBV药物代谢动力学研究表明其具有良好的口服生物利用度[44]。除与特定药物（例如免疫抑制剂-他克莫司）以外，MBV与其他药物相互作用风险较低[41]。

一项Ⅱ期临床试验纳入120例移植后R/R CMV患者，随机分为3个MBV剂量组（400、800和1 200 mg，每日2次），治疗持续24周，结果显示67％的患者在治疗6周内实现CMV血症清除，中位时长为22～28 d，400、800或1 200 mg剂量组CMV血症清除率分别为70％、63％和68％。安全性方面未观察到对骨髓抑制及肾损伤的直接影响[45]。另一项针对R/R CMV感染的Ⅲ期临床试验评估了MBV与传统抗病毒药物（以下1种或多种药物的组合：GCV、VGCV、FOS及CDV，IAT）的有效性。研究结果显示，第8周时，MBV病毒治疗组CMV血症被清除的患者比例为IAT组的2倍以上；且CMV血症清除中位时间较IAT组快5 d。安全性方面，MBV组治疗相关中性粒细胞减少症的发生率更低（MBV组1.7％对GCV/VGCV组25％）；治疗相关急性肾损伤的发生率更低（MBV组1.7％对FOS组19.1％），且无因治疗相关的中性粒细胞减少和急性肾损伤导致停药的病例[45]–[47]。12个月随访结果显示，MBV组患者总死亡率为15.6％（17/109），低于既往报道的接受R/R CMV传统治疗的类似人群的总死亡率[48]–[49]，且无二次移植发生[50]。真实世界研究结果与以上Ⅲ期研究结果一致，治疗第8周时，MBV治疗组CMV血症清除率达66.6％，且未观察到与MBV治疗相关的不良反应[51]。同时，MBV治疗也能减轻患者治疗负担，在调整治疗暴露后，MBV组较IAT组患者住院率降低了34.8％，住院时间缩短了53.8％[52]。以上研究证实MBV对CMV的清除率高、清除速度快、疗效更佳、治疗相关不良反应更小、降低患者死亡率及治疗负担。

2. 来特莫韦（Letermovir, LMV）：来特莫韦目前被批准用于接受HSCT且CMV血清学阳性成年患者CMV感染预防，通过作用于UL56亚基抑制病毒终止酶复合物，从而抑制病毒复制，抗CMV治疗特异性较强[9]。相较于GCV，LMV的骨髓毒性小。但LMV对于抗R/R CMV感染的疗效一直在探索中，目前尚无大样本临床试验结果证实，仅少数病例报道。1例14岁患者接受脐带血HSCT后出现GCV耐药CMV感染，联合使用LMV和FOS后终末器官受累好转，但转换为LMV单药治疗后，CMV血症复发，同时出现GCV、FOS和LMV多重耐药[53]。此病例快速产生LMV耐药性突变可能与LMV耐药屏障弱有关，指南指出，在R/R CMV感染中，尤其是在治疗中枢神经系统疾病时，不建议使用LMV单药治疗[10]。

3. Filociclovir（MBX-400）：Filociclovir是一种核苷类似物，对腺病毒活性和体外CMV耐药病毒株活性有抑制作用[54]–[55]。针对R/R CMV感染的疗效，目前尚无临床数据证实。

4. 布林西多福韦（Brincidofovir, BCV）：BCV是CDV和脂质联合体，为CDV的前体物质，可以竞争性抑制CMV-DNA聚合酶dNTP结合位点，靶向作用于靶细胞，同时延长CDV抗病毒时间[56]。但目前BCV抗CMV感染治疗的临床研究结果尚存在一定争议，El-Haddad等[57]利用BCV联合FOS治疗3例R/R CMV感染的恶性肿瘤患者发现，2例仅伴UL97基因突变的CMV感染患者疗效良好，另外1例伴UL54基因突变患者的CMV血症未能得到控制。由于BCV与CDV通过相同的机制发挥抗CMV作用，因此对于伴UL54基因突变的耐药性CMV感染的疗效欠佳。另外，Ⅲ期SUPPRES研究[56]安全性数据显示口服BCV产生胃肠道不良反应，GVHD发生风险较高，BCV静脉制剂因胃肠道毒性及肾毒性较低受到关注[58]，但能否用于治疗HSCT后R/R CMV感染尚不明确。

（二）免疫治疗

1. IVIG：IVIG的使用存在争议。Erard等[59]的研究显示与单独使用抗CMV药物相比，合并使用IVIG没有观察患者生存的获益。一项Meta分析提示CMV特异性IVIG临床获益证据缺乏[60]。Vassallo等[61]将CMV特异性IVIG用于成功治疗1例CMV视网膜炎的HSCT患者，证实了CMV特异性IVIG和抗病毒药物的联合治疗是安全有效的。Alsuliman等[62]的报道则提示在HSCT后使用CMV特异性IVIG作为挽救治疗有效且安全。因此，对R/R CMV感染疗效仍需探索。

2. 病毒特异性T细胞治疗：过继免疫疗法即重建CMV特异性T细胞反应，过程包括分离、体外培育并向患者输注供者T细胞。随着细胞免疫治疗的开展，CMV感染的高危患者可以通过输注CMV特异性细胞毒性T淋巴细胞（CMV-CTL）获益。我国一项纳入17例替代供者HSCT后R/R CMV感染患者的研究显示过继免疫疗法总有效率为78.95％，提示其可能为R/R CMV感染的又一治疗选择[63]。另一项单中心回顾性研究中纳入单倍体HSCT后CMV感染患者190例，其中难治性CMV感染137例患者接受首次CMV-CTL输注后第6周完全缓解率为86.9％[64]。供者来源或第三方CMV-CTL的安全性和有效性已在非随机临床研究中得到证明，实现了50％～93％的病毒应答，输注相关不良反应及急性GVHD发生率均较低[65]–[66]。CMV-CTL的获取难度、时间成本、经济成本和高剂量类固醇的未知疗效是进行过继免疫疗法的难点。CMV-CTL治疗R/R CMV感染的疗效和安全性尚无大样本、设计良好的随机对照研究结果证实，仍需进一步探索。

除CMV-CTL治疗外，NK细胞疗法和T细胞表面特异受体T细胞疗法也有相关研究报道[67]–[68]。

六、结语

CMV感染是HSCT后最常见的感染之一，目前临床一线抗CMV药物有GCV、VGCV和FOS。然而，仍有一部分患者对传统抗病毒药物治疗出现不应答、不耐受、甚至耐药，进展成为R/R CMV感染，预后较差，是移植后抗CMV感染治疗的难点。现有R/R CMV的治疗方案选择有限，且存在诸多不足，易产生限制治疗的药物毒性，如骨髓抑制和肾毒性，患者迫切需要兼顾病毒清除率高、清除速度快、安全性好的优良方案。针对R/R CMV感染的药物以及免疫治疗仍需进一步探索，获得更多临床证据的支持。
